# Does Physical Self-Concept Mediate the Relationship between Motor Abilities and Physical Activity in Adolescents and Young Adults?

**DOI:** 10.1371/journal.pone.0168539

**Published:** 2017-01-03

**Authors:** Darko Jekauc, Matthias Oliver Wagner, Christian Herrmann, Khaled Hegazy, Alexander Woll

**Affiliations:** 1 Institute for Sport Science, Humboldt University Berlin, Berlin, Germany; 2 Department for Sport Science, University of Konstanz, Konstanz, Germany; 3 Department of Sport, Exercise and Health, University of Basel, Basel, Switzerland; 4 Department of Sports and Sports Science, Karlsruhe Institute of Technology, Karlsruhe, Germany; 5 Faculty of Sport Education Abu Qir, Alexandria University, Alexandria, Egypt; Rutgers University, UNITED STATES

## Abstract

The purpose of this study is to examine the reciprocal relationship between motor abilities and physical activity and the mediation effects of physical self-concept in this relationship using longitudinal data. We expect that the effects of motor abilities on physical activity are rather indirect via physical self-concept and that the effects of physical activity on motor abilities are rather direct without involvement of the motor ability self-concept. Data was obtained from the Motorik-Modul (MoMo) Longitudinal Study in which 335 boys and 363 girls aged 11–17 years old at Baseline were examined twice in a period of six years. Physical activity was assessed by the MoMo Physical Activity Questionnaire for adolescents, physical self-concept by Physical Self-Description Questionnaire and motor abilities by MoMo Motor Test which comprised of the dimensions strength, endurance, coordination and flexibility. Multiple regression analyses were used to analyse the direct and indirect effects. The results of the multiple regression analyses show that the effects of motor abilities on physical activity were only indirect for the dimensions strength, coordination, and flexibility. For the dimension endurance, neither direct nor indirect effects were significant. In the opposite direction, the effects of physical activity on motor abilities were partially mediated by the self-concept of strength. For the dimensions endurance, coordination and flexibility, only indirect were significant. The results of this study support the assumption that the relationship between motor abilities and physical activity is mediated by physical self-concept in both directions. Physical self-concept seems to be an important determinant of adolescents´ physical activity.

## Introduction

In adolescence, physical activity was shown to be inversely related to obesity and positively associated with numerous health benefits as favourable cardiovascular and metabolic disease risk profiles, enhanced bone health and reduced symptoms of depression in children and adolescents [1–4]. Conversely, physical inactivity is associated with increased risks for health impairments such as metabolic syndrome and cardiovascular diseases [5]. However, a large part of the adolescent population is not sufficiently physically active to profit from these health benefits. For example, in Germany only 8% of boys and 5% of girls aged between 14–17 years comply with the recommendation of being moderately to vigorously physically active for at least 60 minutes every day [6]. Similar results for adolescents were also found in other countries [7–11].

The problem of insufficient physical activity seems to exacerbate during adolescence as the proportion of physical active adolescents decreases with increasing age [9,12]. This is not surprising as during adolescence significant and obvious physical and psychological changes occur [13,14]. An important physical activity correlate, during adolescence, is physical self-concept that is also affected by changes in this turbulent life period. It is supposed that physical self-concept during adolescence influences physical activity and sport participation. According to Harterʼs competence motivation theory, competence motivation increases when an individual successfully masters a task [15]. Successful mastering of tasks promotes the perception of self-competence (as well as self-concept) which encourages the person to engage in further activities. This theory has been applied in the context of adolescents’ sport participation and it could be shown that physical self-concept is an important motivation factor for becoming and maintaining being physically active [16].

**Fig 1:**
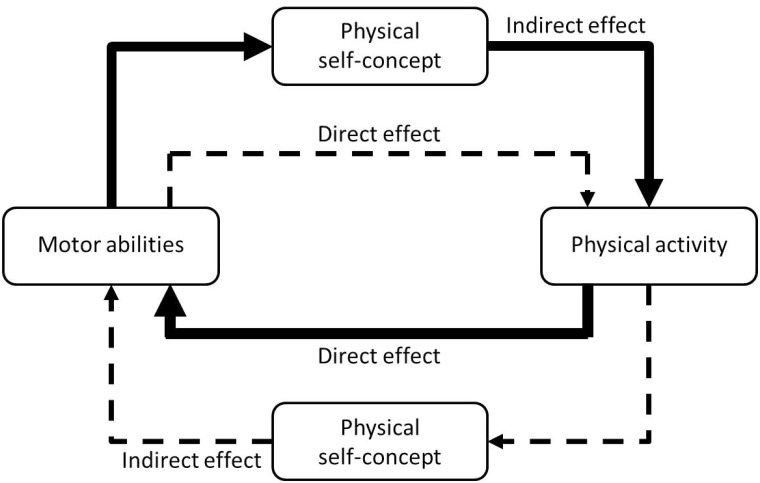
The circular relationship between motor abilities, self-concept and physical activity.

Physical self-concept is assumed to mediate the relationship between physical activity and motor abilities [17,18]. We assume that there is a circular relationship between motor abilities, physical self-concept, and physical activity (see Fig 1). Motor abilities are a source for constructing the own physical self-concept concerning these motor abilities. Well-developed motor abilities lead to good performance in sports and exercise. This implies master experiences and positive feedback from significant others (e.g. trainer, parents, peers) which are related to positive emotions and motivation for physical activity [19,20]. As a consequence, a more positive physical self-concept concerning motor abilities develops. On the contrary, poor developed motor abilities lead to poor performances which entail negative comparisons with peers, lack of master experiences and no positive feedback. As a consequence, negative physical self-concept regarding the motor abilities develops. In this respect, motor abilities represent a source of information for constructing the physical self-concept. As described above, these self-perceptions of motor abilities are the motivating factor for further sport activities. In this regard, it can be assumed that actual motor abilities do not directly influence physical activity and sport participation (dashed line in Fig 1). Instead we suppose that this relationship is mediated by the physical self-concept (solid line in Fig 1). This relationship seems to become stronger with increasing age during adolescence. The stabilization of the self-concept leads to a solidification of this relationship [17]. However, engaging in physical activity and sports also leads to improvements in motor abilities. This is especially true of physical activity occurring in the setting of organised sports, where specific motor skills and abilities are systematically trained, will lead to improvements in motor abilities. In this way, physical activity is a determinant of motor development. Children and adolescents with higher levels of physical activity and sport participation will develop better motor skills and abilities than children and adolescents with low level of physical activity or no participation in sports. Therefore, we believe that the effect of physical activity on motor abilities is rather direct (solid line in Fig 1) and not mediated by the physical self-concept (dashed line in Fig 1). Therefore, we state that the relationship between motor abilities and physical activity is reciprocal and partially mediated by the self-concept (see Fig 1).

According to this hypothesis, there is sound empirical evidence that motor skills [21–26] and motor abilities [27] are related to physical activity. Although the effects are not strong, the evidence is rather consistent [28]. Adolescents with higher levels of physical activity show better performances in motor tests. Furthermore, sound empirical evidence as reported in two reviews support the link between self-perceptions and physical activity [29,30]. Here again, the effects are rather moderate to small. Adolescents with higher scores in self-perceptions are more physically active than adolescents with lower scores. The link between motor skills and abilities on one side and physical self-concept on the other side could also empirically be shown. Evidence was provided that adolescents with better actual performances in motor tests had higher perceptions of their motor abilities [31,32] and skills [33–35]. Adolescents with motor learning disabilities such as developmental coordination disorder also showed lower scores in self-perception of their own abilities than adolescents without developmental coordination disorder [36,37]. However, the mediational effect of self-concept was considerably less the subject of research. To our knowledge only two publications from the same research group explicitly addressed this research question. In a longitudinal study, Barnett et al. [35] found that the perceived sport competence partially mediated the relationship between motor skill proficiency and physical activity. These effects were found only for the object control skills but not for locomotor skills. In another cross-sectional analysis of the same data, Barnett et al. found that the partial mediation effect existed in both directions [18]. However, the reverse pathways were not tested in a longitudinal analysis and a longitudinal analysis would be needed to test whether the mediational effect of self-concept in both directions really exists. Furthermore, the mediational effects were only examined for the subdomain sport competence whereas other subdomains of perceived motor abilities such as coordination, strength or endurance were not examined.

The purpose of this study is to examine the reciprocal relationship between motor abilities and physical activity and the mediation effects of physical self-concept in this relationship using longitudinal data. We expect that the effects of motor abilities on physical activity are rather indirect via physical self-concept and that the effects of physical activity on motor abilities are rather direct without involvement of physical self-concept (see Fig 1).

## Methods

### Subjects and study design

**Fig 2:**
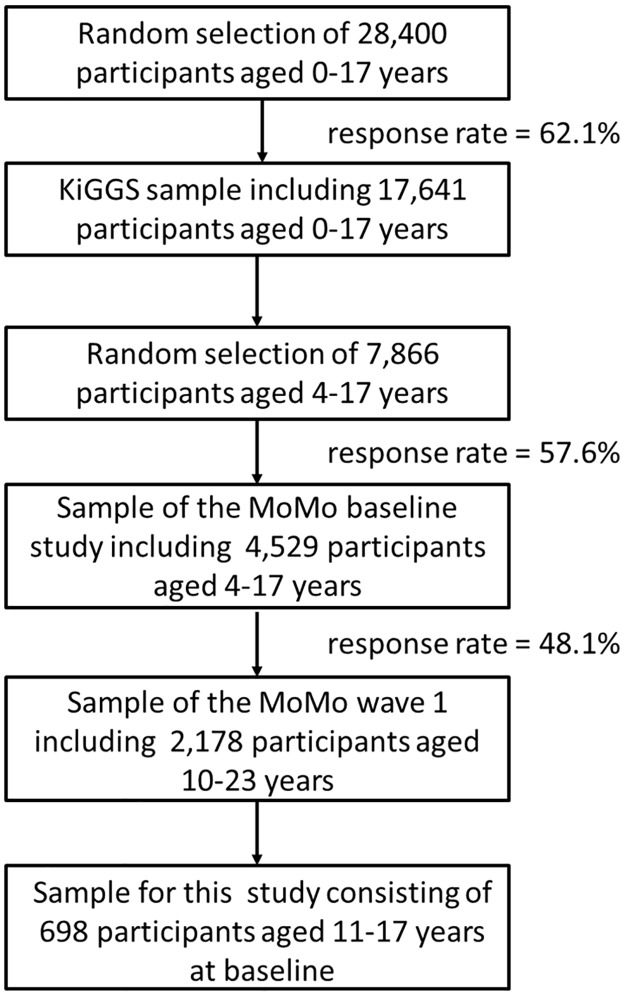
Flow diagram of recruitment.

Data was obtained from the Motorik-Modul (MoMo) Longitudinal Study which aims to examine the prevalence rates and development of physical activity and motor abilities in children, adolescents and young adults in Germany [38]. The MoMo Longitudinal Study is a module of the German Health Interview and Examination Survey (KiGGS) [39]. The baseline of the MoMo Longitudinal Study was conducted between 2003 and 2006 using a nationwide representative sample [40]. The follow-up of the MoMo Longitudinal Study began six years later in September 2009 and ended in July 2012. Participants were recruited using a three step process (see Fig 2). Firstly, a systematic sample of 167 primary sampling units was selected, from an inventory of German communities stratified according to the BIK classification system [39] that measures the level of urbanization and the geographic distribution. The probability of any community being picked was proportional to the number of inhabitants younger than 18 years old. For communities with less than 350 inhabitants under 18 years old, the adjacent community was added to the sample. Secondly, an age stratified sample of randomly selected children and adolescents was drawn from the official registers of local residents for the KiGGS with a total of 28,400 participants aged between 0 and 17 years old [41]. Out of these 28,400 selected participants, 17,641 children and adolescents aged between 0 and 17 years old took part in the KiGGS for a response rate of 62.1%. Thirdly, 7,866 participants aged between 4 and 17 years old in the KiGGS-sample were randomly assigned to the sample of the MoMo-Study. Of these 7,866 participants, 4,529 children and adolescents took part in the MoMo Study at baseline (response rate = 57.6%). The longitudinal sample in the first follow-up included 2,178 participants aged 10–23 years old, which constitutes an overall response rate of 48.1%. For the purposes of this work, only longitudinal data of 335 boys and 363 girls aged 11–17 years old at baseline were included in the analysis (see Table 1). Younger participants were not included in the analysis because a valid and reliable measurement of physical activity could not be ensured for this age group. For the participants that were included, the same measurement procedure was used on both measuring occasions. Detailed information on the data collection techniques and quality of the sample are presented elsewhere [38]. Informed written consent was obtained from the participants and their parents or guardians before the subjects entered into the study according to the Helsinki Declaration. The study was approved by the ethics committee of the Charité, Humboldt University of Berlin.

**Table 1:** Means and standard deviations at baseline and follow-up (N = 698).

Measurement occasion	baseline		follow-up		t-test	
Variable	M	SD	M	SD	t	p
Age (years)	14.2	2.0	20.6	2.0		
PA (MVPA min/week)	110.0	144.9	71.6	126.1	6.2	< .05
Motor abilities						
Strength						
Push-up (in 40 sec)	13.3	3.5	15.1	4.0	-10.7	< .05
Standing long-jump (cm)	166.0	29.8	181.7	37.4	-13.0	< .05
Endurance						
PWC 170 (watt)	111.5	36.9	155.9	59.0	-20.8	< .05
Coordination						
Jumping side-to-side (in 15 sec)	34.2	6.2	39.9	6.8	-19.7	< .05
Single leg stance (contacts in 1 min)	4.5	5.4	2.4	3.8	11.5	< .05
Backward balancing (steps)	34.8	9.2	39.2	8.0	-13.2	< .05
Flexibility						
Forward bending (cm)	-0.3	9.0	1.4	10.0	-6.2	< .05
Self-concept of motor abilities						
Strength			16.9	4.1		
Endurance			16.1	4.7		
Coordination			17.8	3.3		
Flexibility			17.4	3.7		

Note: M = Mean; SD = Standard deviation; PA = Physical activity; MVPA = Moderate to vigorous physical activity;

### Measurement

#### Physical activity

Physical activity was assessed by the MoMo Physical Activity Questionnaire (MoMo-PAQ) for adolescents which measures physical activity in different settings (sports clubs, leisure-time, school, daily activities and overall physical activity) [42]. For this study, we considered only physical activity in sports clubs because we expected that the relationship between physical activity and motor abilities would be highest in this setting and because in Germany a substantial proportion of children’s and adolescent’s physical activity takes place in organised sports clubs [43]. Physical activity in sports clubs was assessed by four items: type, duration, frequency and seasonality of physical activity. By combining these four items, an index was constructed representing the amount of physical activity in sports clubs (minutes of moderate to vigorous physical activity per week). The reliability and validity of the questionnaire are shown elsewhere [42]. The test-retest reliability for one week distance was 0.93 for adolescents aged between 14 and 17 years. Furthermore, the index was significantly correlated with the accelerometer Actigraph GT1M (r = 0.35) and the physical activity diary Previous Day Physical Activity Recall (r = 0.55) [42].

#### Physical self-concept

Physical self-concept was assessed by the German version [44] of the Physical Self-Description Questionnaire [32]. The questionnaire consists of 36 items representing 6 dimensions: strength, endurance, speed, coordination, flexibility, and general athleticism. For the purposes of this work, only strength, endurance, coordination and flexibility were used in order to provide conceptual symmetry with the test of motor abilities. Each dimension was represented by 6 items with a Likert scale ranging between 1 and 6. By summing up the items for each dimension, the range was between 6 and 36. For all dimensions, internal consistency ranged between 0.78 and 0.94 in three different samples of adolescents and young adults [44]. In this sample, internal consistencies were 0.91 for strength, 0.90 for endurance, 0.87 for coordination and 0.88 for flexibility. The results of explorative and confirmative factor analyses provided evidence for the postulated construct validity of the questionnaire [44].

### Motor abilities

Motor abilities were assessed by the physical-fitness test profile. Assessed dimensions were strength (upper and lower limb), endurance (cardiorespiratory fitness), gross motor coordination (dynamic and static balance), and flexibility (trunk).

#### Strength

Dynamic strength of the upper extremities was assessed using the push-up test [45]. Subjects were asked to do as many push-ups as possible within 40sec. Standing long-jumps were used to assess leg power [46]. Maximum distance of a standing long-jump was recorded. The internal consistency of the composite index for strength was 0.67.

#### Endurance

Cardiorespiratory fitness was assessed with the Physical Working Capacity 170 (PWC170) cycle ergometry test (attained watts at 170 beats/min)on an ERG 911S (Ergosana, Bitz, Germany) bicycle [47,48]. Initial workload was calculated as 0.5 watts/kg body mass. The workload was increased incrementally by 0.5 watts/kg body mass every 2 minutes. Subjects continued this progressive protocol until their heart rate (HR) exceeded 190 beats/min for at least 15 seconds, or their pedalling rate was less than 50 revolutions per minute for at least 20 seconds, or until they decided to stop because of exhaustion. HR was measured with a chest-strap T31 monitor (Polar Electro Oy, Kempele, Finland) immediately before each increase in workload. The HR signal was transmitted to the bicycle ergometer. The power in watts generated by a subject at a heart rate of 170 beats/min (PWC170) was obtained by the monitoring investigator’s inter- or extrapolating the measured data in Microsoft Excel.

#### Gross motor coordination

The gross motor coordination was assessed by three items: the jumping side-to-side test, the single leg stance, and the backward balancing. The jumping side-to-side test was used to assess gross motor coordination under time constraint. Subjects were asked to perform as many jumps from side-to-side as possible during two 15-sec intervals within a defined boundary, and the numbers for the two intervals were averaged. Single leg stance was used for assessing gross motor coordination during static precision tasks. Subjects were asked to stand on their dominant leg for one minute with their eyes open, and the number of floor contacts with the contralateral limb was recorded. The backward balancing was based on a body coordination test and allowed the assessment of gross motor coordination during dynamic precision tasks. Subjects were asked to balance backwards on 6 cm, 4.5 cm and 3 cm wide beams, respectively, with two trials per beam. The numbers of steps on each beam were added. The test was terminated if one foot touched the ground. The internal consistency of the composite gross motor coordination index was 0.73.

#### Flexibility

A singular forward bend was used for the assessment of trunk flexibility and the flexibility of the sciatic crural muscle group. The lowest point reached by the fingertips while standing on a box with legs extended was recorded.

### Data analysis

Data was analysed using the Statistical Package for Social Science (SPSS) version 22 (IBM, New York, USA). For bivariate correlations and dependent t-tests Holm-Bonferroni-correction was conducted to rule out the problem of multiple significance tests. The significance level was set a priori at 5%. For motor abilities, raw data was used to calculate means and standard deviations. In order to rule out age and gender effects in regression analyses, all items measuring motor abilities were transformed to standard scores with a mean of zero and standard deviation of one for each sex and age group at baseline. Based on these standard values in each item, composite indices were built for all motor ability dimensions.

Mediation analyses were conducted according to the procedure proposed by Baron and Kenny [49]. In this model, a mediator is a variable that accounts for the relation between the predictor and the criterion. Baron and Kenny stated three conditions which have to be fulfilled to show a mediation effect. Firstly, the independent and dependent variable should be significantly correlated with each other. Secondly, the independent variable and mediator should be significantly correlated. Thirdly, the mediator should be a significant predictor of the dependent variable, whilst controlling for the independent variable. To analyse indirect effects path analyses were conducted based on mediation analyses according to Hayes [50,51]. In each regression analysis, the outcome variable was used from the second measurement occasion (follow-up). Whereas, the initial status of the criterion variable at first measurement occasion (baseline) was used as an additional predictor (covariate). In this way, the confounding effects of initial status according to the autoregression of dependent variable were ruled out. Variables measured at baseline only were used as predictors.

In the first step of the mediation analysis, the initial status of physical activity and motor ability at baseline were used as predictors of self-concept (measured at follow-up). The effects of physical activity and motor ability on the mediator variable were assessed which was necessary to calculate the indirect effects. In the second step, self-concept was included into the regression analysis as an additional predictor (beside physical activity and motor ability at baseline) to predict physical activity at follow-up. Consequently direct effect of motor ability and the effect of mediator variable on physical activity were assessed. In the third step, the outcome variable was motor ability at follow-up, predicted by the same variables as in the second step. Accordingly, the direct effect of physical activity and the effect of the mediator variable could be assessed. In the fourth and fifth step, direct and indirect effects in both directions were analysed. The magnitude of the mediation effect was calculated as the product of both indirect effects. As a result of confidence limits based on the normal distribution for the indirect effects often being found to be inaccurate [52], bootstrap estimation according to Preacher and Hayes was used [53]. In this approach, 1000 bootstrap samples were drawn for the calculation. Based on the bootstrap estimation, 95% confidence intervals for the indirect effects were calculated.

## Results

### Descriptive statistics

Table 1 presents the descriptive statistics for the sample at baseline and follow-up. The results show that the amount of physical activity decreased significantly by 38.4 minutes of moderate-to-vigorous physical activity per week during the period of six years. On the contrary, the performance in motor ability tests for strength, endurance and coordination significantly increased over the same period of time. The strongest increase in performance was observed in the dimension endurance. The performance in flexibility significantly decreased over the period of time. Six years after the first measurement participants of this study were found to be 1.7 cm less flexible. The ratings of self-concept were assessed only at follow-up and mean values vary in the mid-range.

### Strength

**Fig 3:**
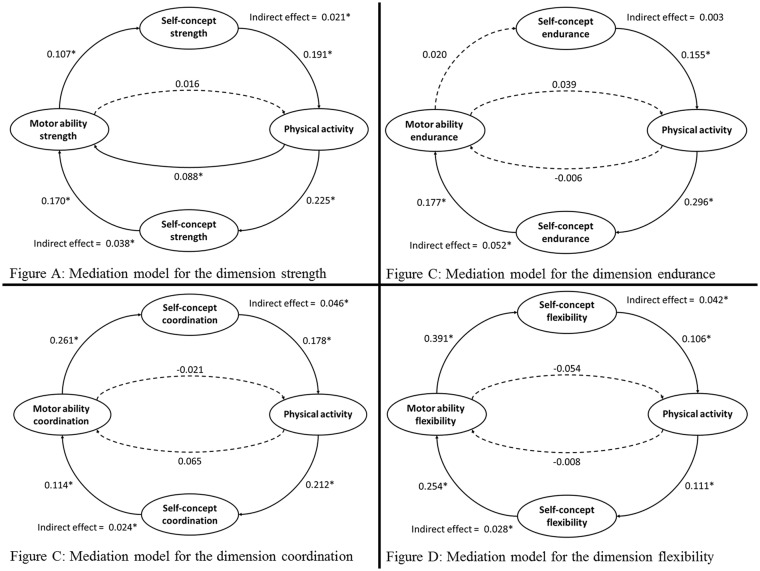
The mediation models.

The results of the multiple regressions are presented in Table 2. A simplified visualization of the results is presented in Fig 3 in Model A. In the first step, physical activity and motor ability strength at baseline were used as predictors of self-concept of strength at follow-up. Both variables could explain 7.4% variance of self-concept of strength. Physical activity (β = 0.225) as well as motor ability strength (β = 0.107) had significant effects on self-concept. In the second step, self-concept was included into the regression analysis as an additional predictor whereas physical activity at follow-up was used as criterion variable. 20.2% of the variance of physical activity at follow-up could be explained by the three variables. Physical activity at baseline (β = 0.356) and self-concept (β = 0.191) had significant unique effects. Motor ability strength did not have a significant effect. In the third step, the outcome variable was motor ability strength at follow-up predicted by the same variables as in the second step. In this regression, 42.2% of the variance of motor ability strength at follow-up could be explained. Motor ability at baseline had the strongest effect (β = 0.567) followed by the self-concept of strength (β = 0.170) and physical activity at baseline (β = 0.088). In the fourth step, it could be shown that the indirect effect of motor ability strength on physical activity via self-concept significantly deviated from zero (β = 0.021) whereas the direct effect was not significant (β = 0.016). In the fifth step, the indirect effect of physical activity on motor ability strength via self-concept also significantly deviated from zero (β = 0.038). However, the direct effect of physical activity on motor ability strength was also significant (β = 0.088).

**Table 2:** Mediation analysis for the dimension strength.

	Regression coefficients					Model summary					
	B	SE	β	t	p	R	R^2^	F	df_1_	df_2_	p
Step 1. Criterion: SC (T2)						0.272	0.074	21.7	2	542	0.000
Intercept	15.871	0.206		77.0	0.000						
PA (T1)	0.006	0.001	0.225	5.3	0.000						
MA (T1)	0.517	0.206	0.107	2.5	0.012						
Step 2. Criterion: PA (T2)						0.449	0.202	45.5	3	541	0.000
Intercept	-57.832	20.515		-2.8	0.005						
SC (T2)	5.926	1.237	0.191	4.8	0.000						
MA (T1)	2.441	5.955	0.016	0.4	0.682						
PA (T1)	0.285	0.323	0.356	8.7	0.000						
Step 3. Criterion: MA (T2)						0.649	0.422	131.5	3	541	0.000
Intercept	-0.757	0.121		6.3	0.000						
SC (T2)	0.037	0.007	0.170	5.0	0.000						
PA (T1)	0.005	0.002	0.088	2.5	0.012						
MA (T1)	0.585	0.035	0.567	16.8	0.000						
	Estimation of direct and indirect effects										
	B	SE	LLCI	ULCI	β						
Step 4. Effect MA on PA											
Direct effect	2.441	5.955	-9.257	14.140	0.016						
Indirect effect	3.061	1.346	0.959	6.238	0.021						
Step 5. Effect PA on MA											
Direct effect	0.005	0.002	0.001	0.009	0.088						
Indirect effect	0.002	0.001	0.001	0.003	0.038						

Note: SC = Self-concept of strength; MA = Motor ability strength; PA = Physical activity; T1 = baseline; T2 = follow-up; B = unstandardized regression coefficient; SE = standard error; β = standardized coefficient; df_1_ = degrees of freedom of the numerator; df_2_ = degrees of freedom of the denominator; LLCI = lower limit of the 95% confidence interval; ULCI = upper limit of the 95% confidence interval.

### Endurance

The results of the multiple regressions for endurance are presented in Table 3 and Fig 3 in Model B. In the first regression, 8.5% of the variance of self-concept endurance could be explained. It could be shown that physical activity (β = 0.296) but not motor ability endurance (β = 0.020) was significantly associated with self-concept of endurance. In the second step, 23.1% of variance of physical activity at follow-up could be explained. Self-concept of endurance (β = 0.155) and physical activity (β = 0.400) but not motor ability endurance (β = 0.039) significantly contributed to the regression. In the third step, 20.7% of the variance of motor ability endurance could be explained. Self-concept (β = 0.177) and motor ability (β = 0.428) had significant unique effects but physical activity (β = -0.006) did not. The results of the fourth step indicated that neither direct (β = 0.039) nor indirect effect (β = 0.003) of motor ability endurance on physical activity was significant. In the fifth step, the results indicated that only the indirect (β = 0.052) but not direct effect (β = -0.006) of physical activity on motor ability endurance was significant.

**Table 3:** Mediation analysis for the dimension endurance.

	Regression coefficients					Model summary					
	B	SE	β	t	p	R	R^2^	F	df_1_	df_2_	p
Step 1. Criterion: SC (T2)						0.292	0.085	10.1	2	418	0.000
Intercept	14.713	0.377		39.0	0.000						
PA (T1)	0.009	0.002	0.296	4.4	0.000						
MA (T1)	0.087	0.286	0.020	0.3	0.761						
Step 2. Criterion: PA (T2)						0.480	0.231	21.7	3	417	0.000
Intercept	-27.225	26.998		-1.0	0.314						
SC (T2)	4.262	1.716	0.155	2.5	0.014						
PA (T1)	0.330	0.053	0.400	6.2	0.000						
MA (T1)	4.628	7.238	0.039	0.6	0.523						
Step 3. Criterion: MA (T2)						0.455	0.207	18.9	3	417	0.000
Intercept	-0.583	0.205		-2.8	0.005						
SC (T2)	0.037	0.013	0.177	2.8	0.006						
MA (T1)	0.376	0.055	0.428	6.8	0.000						
PA (T1)	-0.004	0.004	-0.006	-0.9	0.369						
	Estimation of direct and indirect effects										
	B	SE	LLCI	ULCI	β						
Step 4. Effect MA on PA											
Direct effect	4.628	7.238	-9.637	18.193	0.039						
Indirect effect	0.370	1.163	-1.848	3.006	0.003						
Step 5. Effect PA on MA											
Direct effect	-0.004	0.004	-0.012	0.004	-0.006						
Indirect effect	0.003	0.001	0.001	0.007	0.052						

Note: SC = Self-conc ept of strength; MA = Motor ability strength; PA = Physical activity; T1 = baseline; T2 = follow-up; B = unstandardized regression coefficient; SE = standard error; β = standardized coefficient; df_1_ = degrees of freedom of the numerator; df_2_ = degrees of freedom of the denominator; LLCI = lower limit of the 95% confidence interval; ULCI = upper limit of the 95% confidence interval.

### Coordination

The results of the multiple regressions for coordination are presented in Table 4 and Fig 3 in Model C. In the first step, 13.8% of the variance of self-concept for coordination was explained. Physical activity (β = 0.212) as well as motor ability coordination (β = 0.261) had significant effects on self-concept. In the second step, 19.7% of the variance of physical activity at follow-up could be explained. Self-concept of coordination (β = 0.178) and physical activity (β = 0.368) but not motor ability coordination (β = -0.021) had significant effects. In the third step, the regression explained 19.2% of the variance of motor ability coordination at follow-up. Self-concept of coordination (β = 0.114) and motor ability at baseline (β = 0.366) but not physical activity (β = 0.065) significantly contributed to the regression. In the fourth step, it could be shown that only the indirect (β = 0.046) but not the direct effect (β = 0.021) of motor ability coordination on physical activity was significant. In the fifth step, the results revealed that again only the indirect (β = 0.024) but not the direct (β = 0.065) effect significantly deviated from zero.

**Table 4:** Mediation analysis for the dimension coordination.

	Regression coefficients					Model summary					
	B	SE	β	t	p	R	R^2^	F	df_1_	df_2_	p
Step 1. Criterion: SC (T2)						0.371	0.138	43.6	2	547	0.000
Intercept	16.962	0.165		102.6	0.000						
PA (T1)	0.005	0.001	0.212	5.2	0.000						
MA (T1)	1.110	0.174	0.261	6.4	0.000						
Step 2. Criterion: PA (T2)						0.444	0.197	44.7	3	546	0.000
Intercept	-74.742	26.234		-2.8	0.005						
SC (T2)	6.497	1.508	0.178	4.3	0.000						
PA (T1)	0.288	0.032	0.368	9.1	0.000						
MA (T1)	-3.271	6.346	-0.021	-0.5	0.606						
Step 3. Criterion: MA (T2)						0.438	0.192	43.1	3	546	0.000
Intercept	-0.318	0.102		-3.1	0.002						
SC (T2)	0.016	0.006	0.114	2.7	0.006						
MA (T1)	0.221	0.025	0.366	8.9	0.000						
PA (T1)	0.020	0.012	0.065	1.6	0.110						
	Estimation of direct and indirect effects										
	B	SE	LLCI	ULCI	β						
Step 4. Effect MA on PA											
Direct effect	-3.271	6.346	-15.736	9.193	0.021						
Indirect effect	7.213	2.037	3.973	12.211	0.046						
Step 5. Effect PA on MA											
Direct effect	0.020	0.012	-0.004	0.044	0.065						
Indirect effect	0.007	0.003	0.002	0.014	0.024						

Note: SC = Self-concept of strength; MA = Motor ability strength; PA = Physical activity; T1 = baseline; T2 = follow-up; B = unstandardized regression coefficient; SE = standard error; β = standardized coefficient; df_1_ = degrees of freedom of the numerator; df_2_ = degrees of freedom of the denominator; LLCI = lower limit of the 95% confidence interval; ULCI = upper limit of the 95% confidence interval.

### Flexibility

The results of the multiple regressions for flexibility are presented in Table 5 and Fig 3 in Model D. In the first step, 18.5% of the variance of self-concept flexibility was explained. Physical activity (β = 0.111) as well as motor ability flexibility (β = 0.391) had a significant unique effect on self-concept of flexibility. In the second step, 17.2% of the variance of physical activity at follow-up could be explained. Self-concept of flexibility (β = 0.106) and physical activity at baseline (β = 0.394) but not motor ability flexibility (β = -0.054) significantly contributed to the regression. In the third step, 56.6% of the variance of motor ability flexibility at follow-up was explained. Self-concept (β = 0.254) and motor ability at baseline (β = 0.612) but not physical activity (β = -0.008) had significant unique effects. In the fourth step, it could be shown that only the indirect (β = 0.042) but not the direct (β = -0.054) effect of motor ability flexibility on physical activity significantly deviated from zero. Again in the fifth step, the indirect (β = 0.028) but not the direct (β = -0.008) effect of physical activity on motor ability flexibility significantly deviated from zero.

**Table 5:** Mediation analysis for the dimension flexibility.

	Regression coefficients					Model summary					
	B	SE	β	t	p	R	R^2^	F	df_1_	df_2_	p
Step 1. Criterion: SC (T2)						0.430	0.185	64.2	2	566	0.000
Intercept	16.763	0.184		91.297	0.000						
PA (T1)	0.003	0.001	0.111	2.9	0.004						
MA (T1)	1.466	0.146	0.391	10.0	0.000						
Step 2. Criterion: PA (T2)						0.414	0.172	39.1	3	565	0.000
Intercept	-21.640	24.485		-0.9	0.377						
SC (T2)	3.532	1.413	0.106	2.5	0.013						
PA (T1)	0.325	0.033	0.394	10.0	0.000						
MA (T1)	-6.787	5.333	-0.054	-1.3	0.204						
Step 3. Criterion: MA (T2)						0.752	0.566	245.4	3	565	0.000
Intercept	-1.147	0.139		8.2	0.000						
SC (T2)	0.067	0.008	0.254	8.3	0.000						
MA (T1)	0.602	0.030	0.612	19.8	0.000						
PA (T1)	-0.005	0.018	-0.008	-0.3	0.775						
	Estimation of direct and indirect effects										
	B	SE	LLCI	ULCI	β						
Step 4. Effect MA on PA											
Direct effect	-6.787	5.333	-17.267	3.689	-0.054						
Indirect effect	5.179	2.238	1.073	9.888	0.042						
Step 5. Effect PA on MA											
Direct effect	-0.005	0.018	-0.042	0.031	-0.008						
Indirect effect	0.018	0.007	0.005	0.033	0.028						

Note: SC = Self-concept of strength; MA = Motor ability strength; PA = Physical activity; T1 = baseline; T2 = follow-up; B = unstandardized regression coefficient; SE = standard error; β = standardized coefficient; df_1_ = degrees of freedom of the numerator; df_2_ = degrees of freedom of the denominator; LLCI = lower limit of the 95% confidence interval; ULCI = upper limit of the 95% confidence interval

## Discussion

The main assumption of this study was that the effects of motor abilities on physical activity would be mediated by self-concept and would not be direct (see Fig 1). In the opposite direction, we assumed that the effects of physical activity on motor abilities would be rather direct and not mediated by physical self-concept. This research question was examined in four specific domains: strength, endurance, coordination, and flexibility.

### Strength

In the domain strength, the mediation effects of self-concept could be found in both directions. The effect of the motor ability strength on physical activity was only indirect. This indicates that well developed strength is associated with a more positive self-concept which leads to increased physical activity. The direct effect of motor ability is, however, not significant meaning that strength in our study does not per se influence the development of physical ability. Contrary to our assumption, the mediation effect was also found in the opposite direction. However, the direct effect of physical activity on motor ability was also significant and stronger than the indirect effect. This result indicates that physical activity primarily has a direct impact on strength but there also exists an indirect effect via self-concept.

### Endurance

In the domain endurance, the effect of motor ability on physical activity is neither direct nor indirect. This result is not in accordance with our hypothesis as it would mean that motor ability endurance is not important for future physical activity. However, the self-concept of endurance is significantly associated with physical activity indicating that self-concept of endurance might be an important determinant of physical activity. Furthermore, the self-concept of endurance mediates the effects of physical activity on motor ability endurance. This finding does not correspond with our initial hypothesis but can be logically explained when considering that the level of physical activity decreases in the period of transition from adolescence to young adulthood. This reduction in physical activity does not necessarily lead to a deterioration of motor ability endurance because the physical development in this period of life strongly influences motor ability endurance and compensates for the effects of reduced physical activity. The performance in the motor test for endurance increases from baseline to follow-up by circa one standard deviation. This development of motor ability endurance is larger than the development of other motor abilities in this period of life. Therefore, the relationship between motor ability endurance and physical activity might be attenuated. However, physical activity influences the self-concept of endurance which in turn is related to the motor ability endurance. This shows how, the indirect effect of physical activity on motor ability endurance via the self-concept can be explained.

### Coordination

In the domain coordination, the indirect effect of the motor ability coordination on physical activity is significant whereas the direct effect is not. These results comply with our hypothesis meaning that well developed coordination positively influences the future self-concept of coordination. Furthermore, the self-concept of coordination seems to be important for future physical activity. Accordingly, the effect of the motor ability coordination on future physical activity is mediated by the self-concept of coordination. Motor ability coordination does not per se influence physical activity but only indirectly via self-concept. However, the indirect effect in the contrary direction is also significant and the direct effect is not. This fact is not in accordance with our assumption. We expected that the effect of physical activity on motor ability coordination would not be mediated but rather direct. It seems that physical activity is an important source for a positive self-concept of coordination and self-concept of coordination is related to motor ability coordination.

### Flexibility

In the domain flexibility, the constellation of results is the same as in the domain coordination. The indirect effects are significant in both directions and direct effects are not. These findings also partially support our assumptions. The indirect effect of motor ability flexibility on physical activity is in accordance with our theoretical position. Having a flexible body leads to a positive self-concept of flexibility which in turn influences future physical activity. Contrary to our assumptions, being physical active in a sports club leads to a positive self-concept of flexibility which in turn influences motor ability flexibility.

### General discussion

The results of our study partially support our assumptions. In three domains, the effects of motor abilities on physical activity were mediated by self-concept whereas the direct effects were not significant meaning that there was a full mediation. Similar results were found by Barnett and colleagues [18] who found that the effects of locomotor skills on physical activity were fully mediated by perceived sport competence. These findings are congruent with the theoretical positions of Harter, Weiss, and colleagues [15,54,55]. According to these theories, the mastery experiences lead to a positive self-concept, which is an essential component for maintaining physical activity in children and adolescents. These positive experiences and the related positive self-concept seem to be especially important in the period of transition from adolescence to young adulthood as in this period the amount of physical activity decreases and many individuals stop being physically active in sports clubs [43]. In this developmental period, changes in self-concept occur which are important for future psychological development of the individual. Motivational strategies to increase positive perceptions of self could be important to increase the maintenance of physical activity in the transition from adolescence to young adulthood.

However, results from the analysis in the opposite direction contradict our assumptions. We expected to find only direct but not indirect effects of physical activity on motor abilities. Instead, we found significant indirect effects in all four domains and the direct effect was only significant in the domain of strength. However, these results are congruent with the findings of the study conducted by Barnett and colleagues [18]. In this study, the researchers found that the effects of physical activity on locomotor skills are also fully mediated by perceived sport competence. These results mean that physical activity is an important source of information for shaping the physical self-concept. Adolescents seem to use the experiences made during physical activity to form their self-concept. Furthermore, this self-concept is positively related to motor abilities. This finding can only be explained by an expectation effect in which a positive self-concept is associated with elevated expectations which in turn positively influence performance in motor tests. However, the direct effects of physical activity on motor abilities might be attenuated over a period of six years as in this stage of life great developments in motor abilities occur and the stability of physical activity is rather low [56].

In general, it can be stated that the effects in both directions were rather small. For a shorter period of time, greater effects could be expected. It is possible that in our model some unknown theoretical mechanisms or methodological issues (e.g. long distance between measurement occasions) exist which could explain these findings. Therefore, further studies are needed to clarify these theoretical and methodological issues.

This study has several merits and limitations. It is based on a nationwide representative sample of adolescents and young adults in Germany. The sample is sufficiently large to detect even small effects. The longitudinal data used in this work provide stronger evidence for causal effects than cross-sectional data. Direct as well as indirect effects were controlled for the stability of the predicted variable. In this way, spuriousness could be reduced. However, self-concept was measured only at follow-up but not at baseline. Therefore, the changes of self-concept over time cannot be evaluated. An analysis of the differences in self-concept would provide an even deeper understanding of the developmental issues. Furthermore, the measurement of endurance based on PWC170 might not be the most reliable and valid measurement method of endurance to date. Therefore, the findings in the domain endurance should be considered with caution. The lag between the measurement occasions might have been too long to soundly investigate the mechanisms of mediations. Therefore, further studies with closer time frames between measurements would increase the accuracy of the investigation. Finally, physical activity was measured by subjective measurement methods which were shown to have limited reliability and validity [57]. It is possible that objective measurement methods of physical activity would yield larger effects than found in this study.

## Conclusions

This study provides insights about role the physical self-concept as a predictor and mediator in the relationship between physical activity and motor abilities. The results of this study support the assumption that the effects of motor abilities on physical activity are not direct but rather mediated by self-concept. Self-concept seems to be an important determinant of adolescents´ physical activity. Especially in the transition period between adolescence and young adulthood, interventions aiming to increase positive self-concept are promising. However, the effects of physical activity on motor abilities are also partially indirect and mediated by self-concept and that is not compatible with our theoretical considerations. Further studies are needed to resolve theoretical and methodological issues.
